# Effects of one year of extreme isolation in Antarctica on olfactory and gustatory functions

**DOI:** 10.1038/s41598-025-16900-x

**Published:** 2025-09-05

**Authors:** Bea Klos, Sophia Wolf, Kathrin Ohla, Stijn Thoolen, Hannes Hagson, Paul Enck, Isabelle Mack

**Affiliations:** 1 Internal Medicine VI, Psychosomatic Medicine and Psychotherapy, University Hospital Tübingen, Tübingen, Germany; 2 Science & Research, dsm-firmenich, Satigny, Switzerland; 3 French Polar Institute Paul-Émile Victor - Sponsored by the European Space Agency, Brest, France; 4 Department of Psychiatry, Massachusetts General Hospital, Harvard Medical School, Boston, MA USA

**Keywords:** Isolated confined extreme environments, Olfactory function, Gustatory function, Hyposmia, Hypogeusia, Space analog, Environmental impact, Respiration, Digestive signs and symptoms, Hypoxia, Malnutrition, Gastroenterology

## Abstract

Taste and smell are critical for food intake and maintaining adequate energy balance, particularly in isolated, confined, and extreme (ICE) environments. Hypoxic conditions, low humidity, and limited chemosensory exposure at Concordia Station in Antarctica may impair taste and smell functions, though research remains scarce. Gustatory and olfactory functions were assessed in 19 participants (39.2 ± 10.9 years) during two overwintering missions at Antarctic Concordia Station. Testing occurred six weeks pre-departure, three times during isolation, and six months post-isolation. Gustatory function was evaluated using ODOFIN Taste Strips; olfactory function using ODOFIN Sniffin’ Sticks. Additionally, subjective sensory reports were collected. Hyposmia increased during isolation, accompanied by a trend toward declining smell identification (*p* = 0.054), with limited follow-up data offering no clear evidence of recovery. Hypogeusia was primarily reflected in an elevated prevalence of reduced salt sensitivity during mid- to late isolation (*p* = 0.036), returning to baseline levels post-expedition. Subjective evaluations only partially aligned with psychophysical test results. A one-year stay at Concordia Station revealed individual variability in chemosensory responses, highlighting the sensitivity of taste and smell to environmental extremes. While causality remains unclear, the findings emphasize the need to monitor chemosensory function in extreme settings and constrained environments in everyday life.

**Supplementary Information:**

The online version contains supplementary material available at 10.1038/s41598-025-16900-x.

## Introduction

Ensuring sufficient energy and nutrient intake is a critical challenge in isolated, confined, and extreme (ICE) environments^1–3^, where environmental stressors can impact physiological and psychological well-being. Despite adequate food availability^4^, individuals in ICE settings often fail to meet their daily caloric requirements^3,5,6^, leading to weight loss and potential nutrient deficiencies^6–9^. These deficits are particularly concerning during long-duration stays^10–14^, where maintaining physical and cognitive function is essential^3^.

Among several potential contributors, alterations in taste and smell are suggested to affect dietary choices and food intake in ICE environments^15^, though their exact impact remains unclear. Taste perception plays a crucial role in nutrient detection, meal completion, and satiety^16^. It encompasses hedonics and at least five basic qualities sweet, sour, salty, bitter, and umami^17^, which regulate dietary intake^16^. The olfactory system contributes to food perception and thus plays a significant role in appetite regulation, food preferences, and avoidance behaviors^16^. Orthonasal odors stimulate appetite and shape dietary choices^16^, while also detecting dangers, and supporting social communication.

In ICE environments, such as space missions, polar stations, or isolated confined controlled environments like bed rest studies, anecdotal reports confirm altered taste and smell perceptions^15,18–20^. However, experimental findings from ICE environments remain inconsistent, highlighting the disparity between subjective perception and measured chemosensory performance. Findings range from overall^18,21–25^ or selective declines^26–29^ in sensory function to stable performances ^26,28,30^ or even improvements^18,31^, possibly reflecting the variability in environmental stressors, exposure durations, and methodological frameworks across studies.

The Concordia Research Station in Antarctica provides a unique opportunity to examine chemosensory function in a high-altitude ICE environment characterized by chronic hypobaric hypoxia, extreme cold, low humidity, circadian disruption, and prolonged isolation^32^. Crews frequently report nasal congestion, dryness, and sore throat^33^, symptoms often linked to reduced nasal airflow and impaired olfactory function. Similar symptoms and chemosensory impairments have also been found in other high-altitude research settings^24,25,27–29^, where hypoxia, cold, and isolation create environmental conditions partially comparable to those at Concordia. However, findings across studies remain inconsistent, and longitudinal data from environments that combine multiple ICE-related stressors are scarce, leaving key aspects of chemosensory adaptation poorly understood. Disparities between subjective reports and objective measurements further complicate interpretation.

This study addresses these gaps by examining how prolonged exposure to an ICE environment affects olfactory and gustatory function. Changes were assessed before (T0, Pre), during (T1, February; T2, June; T3, October), and after (T4, Post) a one-year stay at Concordia Station in Antarctica. In addition to mapping changes in sensory performance over time, the relationship between smell and taste were explored and discrepancies between subjective reports and psychophysical test outcomes were examined.

By focusing on these questions, the study advances our understanding of human sensory adaptation under extreme conditions. These insights are not only relevant for sustaining well-being and performance in remote expeditions and long-duration spaceflights but may also inform how sensory perception is affected by isolation, monotony, and altered environments in everyday life—such as in long-term care, during pandemic lockdowns, or other socially constrained settings.

## Results

### Study population

The final analysis included 19 Caucasian participants (84.2% male; mean age: 39.2 ± 10.9 years). The baseline characteristics are presented in Table 1.

**Table 1:** Baseline characteristics of 19 participants are presented as mean ± standard deviation (M ± SD) for age and BMI. Nominal variables, including winter-over period, sex, smoking and allergies, are reported as absolute frequencies (N) and percentages (%). ^1^Specific allergies include cats, pollen, house dust, mites, and penicillin.

Baseline sample characteristics	
**Age** (years)	39.2 ± 10.9
**Overwintering period N (%)**	
2019/2020	10 (52.6%)
2021/2022	9 (47.4%)
**Sex distribution N (%)**	
Female	3 (15.8%)
Male	16 (84.2%)
**BMI (kg/m**^2^**)**	26.40 ± 3.04
**Smoking N (%)**	
No	16 (84.2%)
Daily	3 (15.8%)
**Allergies N (%)**	
None	14 (73.7%)
Specific allergies^1^	5 (26.3%)

BMI, Body mass index.

At baseline, participants had an average body mass index (BMI) of 26.40 ± 3.04 kg/m^2^. By the end of the Antarctic stay, the mean BMI had decreased by 1.09 units to 25.31 ± 3.37 kg/m^2^ but had increased by 1.66 units at the 6-month follow-up to 26.97 ± 3.13 kg/m^2^, exceeding baseline levels. Most participants were non-smokers at baseline (84.2%). During isolation, daily smoking declined, with 15.8% reporting occasional use. At follow-up, daily smoking returned to 15.8%, and occasional smoking dropped to 10.5%. All assessments were conducted by one of two trained study physicians, each responsible for all repeated measurements within a given study campaign. The physicians were not part of the study sample.

### Hyposmia was frequently associated with taste-related issues, particularly those involving salty taste

**Fig. 1:**
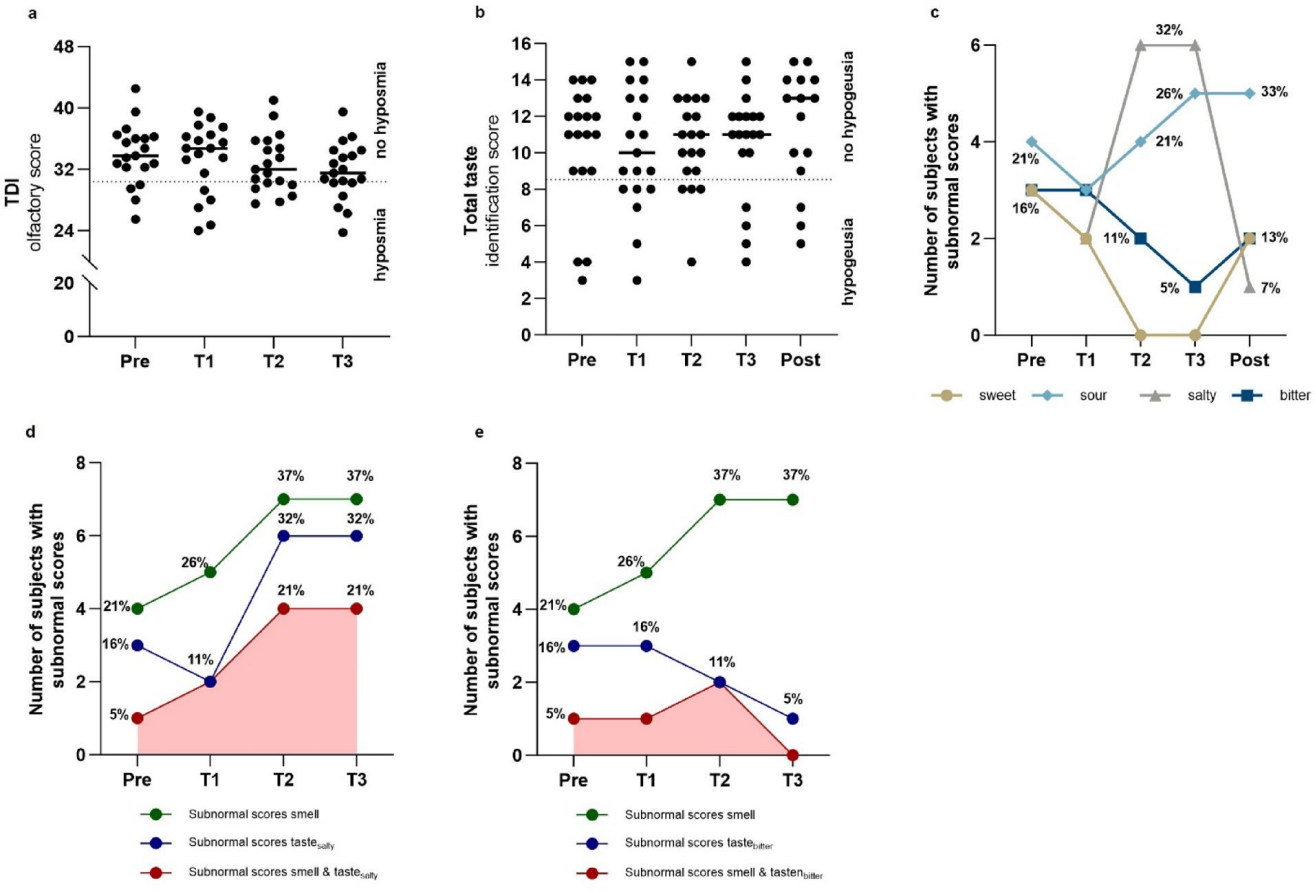
Prevalence of hyposmia, hypogeusia, partial hypogeusia and their combined occurrence. Prevalence of hyposmia (**a**), hypogeusia (**b**), partial hypogeusia (**c**), and their combined occurrence (**d**, **e**) before isolation (*Pre,* N = 19), during isolation (*T1* = February, *T2* = June, *T3* = October, N = 19), and six months post-isolation (*Post,* n = 15). (**a**) Threshold, Discrimination and Identification (TDI) score range: 0–48; hyposmia: 16.25–30.5; normosmia: ≥ 30.75. (**b**) Taste score range: 0–16; hypogeusia: < 9; normogeusia: ≥ 9. (**c**) Subnormal: < 2 (sweet/sour/salty), < 1 (bitter); shown as counts and %. (**d**) Co-occurrence of hyposmia and subnormal scores for salty (%). (**e**) Co-occurrence of hyposmia and subnormal scores for bitter (%).

At baseline, 4 of 19 participants (21%) were classified as hyposmic, which is slightly above the expected 17% for comparable populations^34^. Hyposmia prevalence increased to 5/19 (26%) at T1 and peaked at 7/19 (37%) at T2 and T3 (Fig. 1A), though these increases were not statistically significant (Cochran’s Q = 2.2, *p* = 0.534), likely because individual trajectories were highly variable over time (Supplementary Figure S1a). T4 was excluded from analysis due to limited participation in olfactory testing (n = 5).

Hypogeusia was present in 3/19 participants (16%) at baseline, which is 10% higher than expected in healthy reference populations^35^. During the Antarctic stay, hypogeusia prevalence peaked at T1 with 6/19 (32%) and decreased and stabilized at T2 and T3 with 4/19 (21%, Cochran’s Q = 2.5, *p* = 0.479). Post-isolation, 3/15 (20%) showed hypogeusia (Fig. 1B; Cochran’s Q = 1.9, *p* = 0.760). In line with olfactory impairments, hypogeusia varied across individuals and did not follow a consistent pattern over time (Supplementary Figure S1b).

To further examine these gustatory impairments, subnormal scores for individual taste qualities were analyzed for partial hypogeusia (Fig. 1C). At baseline, 3/19 participants (16%) had subnormal scores for sweet, salty, and bitter tastes, whereas 4/19 (21%) were considered subthreshold for sour. Bitter and sweet scores improved over time, while sour and salty perception worsened, with subnormal salty scores doubling at T2 and T3 (6/19; 32%). Notably, post-isolation, salty scores improved (1/15; 7%), but subnormal sour scores persisted (5/15; 33%). Although no statistically significant differences were found in the prevalence of subnormal scores over time, a decreasing trend was observed for subnormal salty scores across T0–T4 (n = 15; Cochran’s Q = 8.2, *p* = 0.085; Fig. 1C).

Co-occurrence of hyposmia and hypogeusia was observed in one participant at T0, in two at T1, and in three at both T2 and T3 (Cochran’s Q = 1.4, *p* = 0.697), as reflected in a medium correlation between overall taste and smell scores at T2 (ρ = 0.479, *p* = 0.038). Hyposmia co-occurred with subnormal salty scores (Fig. 1D), particularly at T2 and T3 (four cases each). However, significant correlations between salty taste and olfactory function were only observed at T3: salty taste scores at this time point were strongly associated with the overall TDI score (sum score for smell threshold, discrimination and identification subscores; ρ = 0.542, *p* = 0.016) and moderately correlated with the smell identification subscore (ρ = 0.461, *p* = 0.047). Furthermore, both participants with bitter taste dysfunction at T2 also exhibited hyposmia (Fig. 1E). This co-occurrence was supported by a moderate correlation between TDI and bitter identification scores at T2 (ρ = 0.497, *p* = 0.030), and a positive correlation between odor discrimination and the bitter scores (ρ = 0.596, *p* = 0.007). Supplementary Table S1 contains the full set of correlation results across time points.

Although salty taste exhibited the highest prevalence of subnormal scores, the highest concentration of bitter and sour taste strips was not identified by up to 26.3% of participants. Nevertheless, no statistically significant differences emerged in identification performance at the highest concentration across taste qualities, with recognition rates remaining relatively stable (sweet: 94.7–100% (Cochran’s Q = 4.4, *p* = 0.223); sour: 73.7–84.2% (Cochran’s Q = 1.4, *p* = 0.709); bitter: 73.7–84.2% (Cochran’s Q = 1.6, *p* = 0.666); salty: 78.9–84.2% (Cochran’s Q = 1.1, *p* = 0.771)). Complete failures to identify a taste at any tested concentration, indicating no recognition across all levels, were documented for bitter (up to 15.8%), salty (up to 10.5%), and sour (up to 5.3%), while sweet was consistently identified at least at one concentration throughout all time points.

Taste confusion patterns aligned with previous reports^36^. Bitter was often undetected, while sour and salty were frequently confused, suggesting overlapping perception. Sweet was occasionally misidentified, but errors seemed random. Most identification errors occurred at low stimulus intensity, likely reflecting reduced sensory input to the central nervous system.

### Smell function and salty taste progressively declined during isolation

**Fig. 2:**
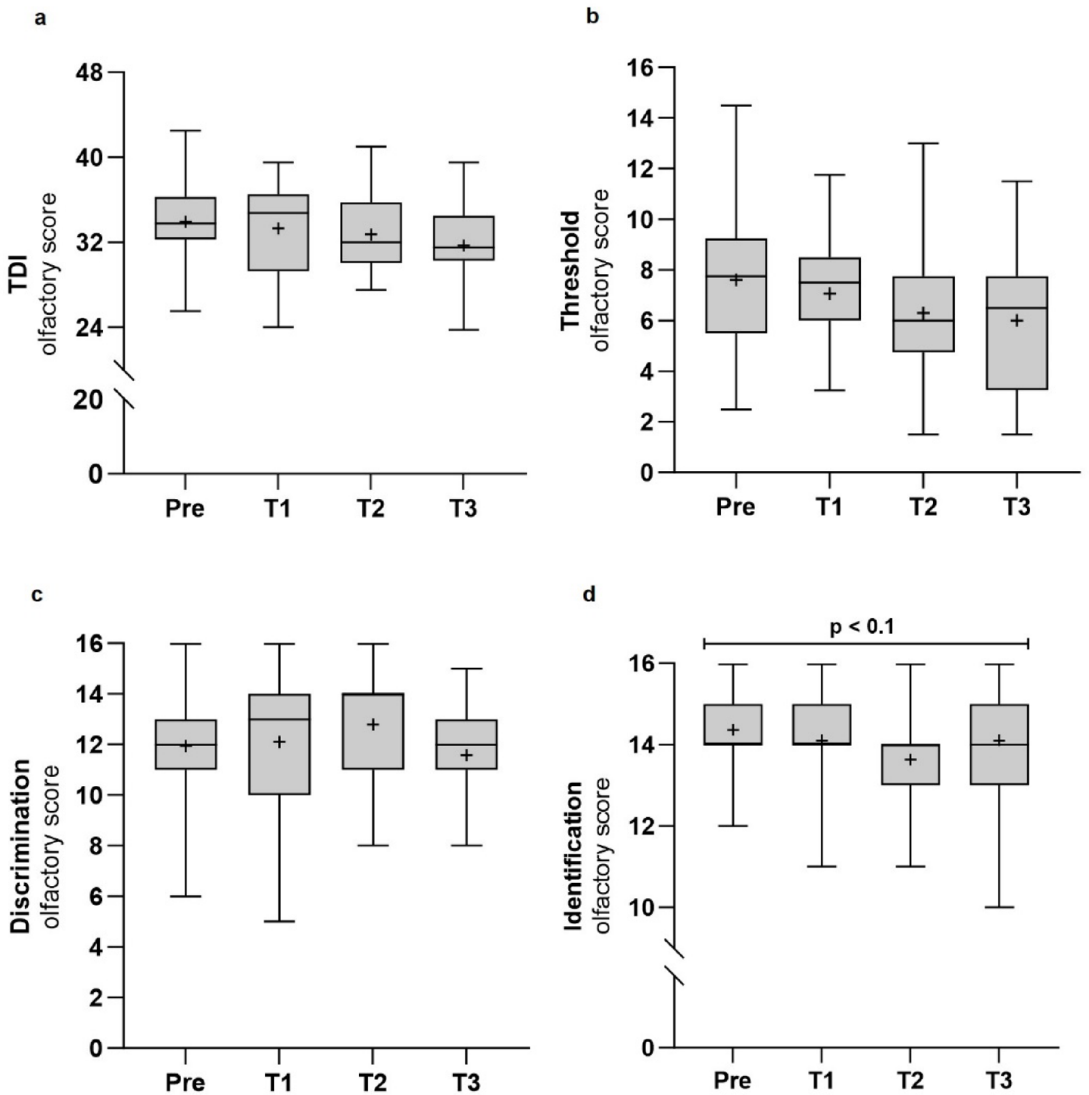
Olfactory performance over time. Test scores for overall smell score (combined Threshold, Discrimination and Identification (TDI) score) (**a**) with odor threshold (**b**), discrimination (**c**), identification subscores (**d**) were assessed before (*Pre,* N = 19) and during isolation (*T1* = February, *T2* = June, *T3* = October, N = 19). Data shown as boxplots: median (─), mean (+), interquartile range (box), minimum (⟘), and maximum (⟙). Max scores: 16 per subtest, 48 total. Statistical trend: 0.05 ≤ *p* < 0.1.

In line with the data for hyposmia, the smell identification scores tended to decrease over time (χ^2^(3) = 7.65, *p* = 0.054, Fig. 2A), indicating a progressive impairment in the ability to identify familiar odors. However, the trend was not supported by the Bayes factor (BF_10_ = 0.472). Changes in TDI score (F(2.11,37.95) = 1.92, *p* = 0.158, BF_10_ = 0.522), threshold (F(3,54) = 1.95, *p* = 0.132, BF_10_ = 0.600) and discrimination subscores (χ^2^(3) = 3.45, *p* = 0.327, BF_10_ = 0.360) over time did not reach statistical significance (Fig. 2B–D). An exploratory subgroup analysis (n = 5) was conducted to evaluate changes in olfactory function six months post-isolation (Supplementary Figure S2). TDI scores declined significantly from T0 to T4 (F(4,16) = 5.94, *p* = 0.004, BF_10_ = 10.871), along with reductions in threshold (F(4,16) = 5.63, *p* = 0.005, BF_10_ = 11.904) and identification subscores (χ^2^(4) = 10.36, *p* = 0.035, BF_10_ = 2.736), indicating a progressive decline in olfactory sensitivity and identification ability over time. Comparable TDI scores at T3 and T4 suggest persisting olfactory impairment post-isolation.

**Fig. 3:**
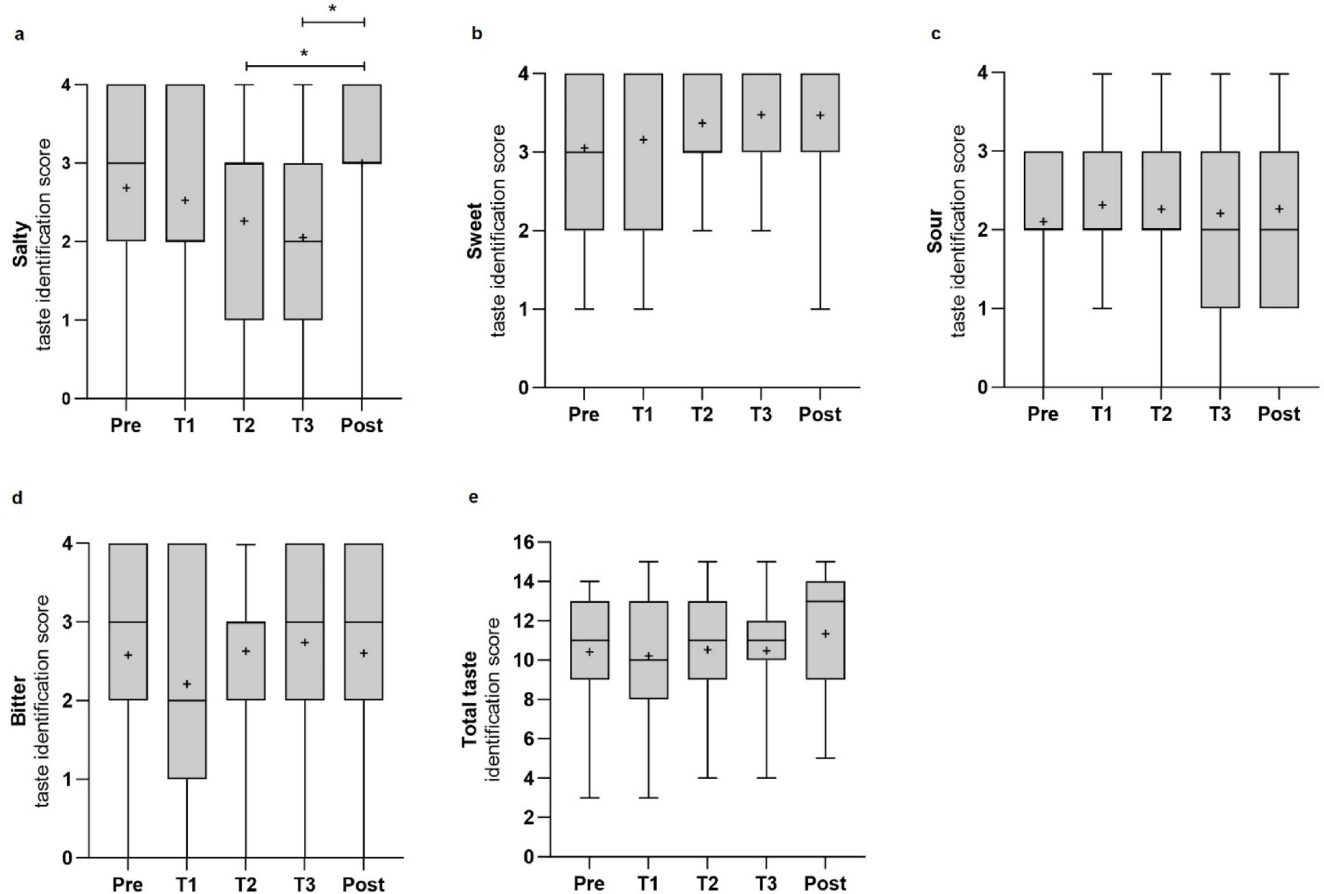
Gustatory performance over time. Taste identification scores are shown for salty (**a**), sweet (**b**), sour (**c**), bitter (**d**), and the total taste score (**e**) before isolation (*Pre,* N = 19), during isolation (*T1* = February, *T2* = June, *T3* = October, N = 19), and six months post-isolation (*Post,* n = 15). Data shown as boxplots: median (─), mean (+), interquartile range (box), minimum (⟘), and maximum (⟙). Significant differences (*p* < 0.05) are marked (*). Max scores: 4 per subtest, 16 total.

Consistent with the overall pattern of hypogeusia—especially the increased occurrence of partial hypogeusia for salty taste—the salty identification score showed a significant change over time (n = 15, χ^2^(4) = 10.283, *p* = 0.036), though Bayesian analysis provided only weak evidence for this effect (BF_10_ = 1.047; Fig. 3A). Post hoc tests identified a significant reduction in salty taste identification scores at T2 compared to T4 (*p* = 0.033) and at T3 compared to T4 (*p* = 0.028), aligning with the upward trend observed in the prevalence rates of partial hypogeusia for salty taste (Fig. 1C). In contrast, no significant changes over time were observed in the identification scores for sweet (χ^2^(4) = 3.12, *p* = 0.538, BF_10_ = 0.095), sour (χ^2^(4) = 1.16, *p* = 0.884, BF_10_ = 0.074), or bitter tastes (χ^2^(4) = 3.19, *p* = 0.527, BF_10_ = 0.141), nor in overall taste identification (χ^2^(4) = 0.57, *p* = 0.966, BF_10_ = 0.071, Fig. 3B–E).

A detailed summary of outcomes across all timepoints is presented in Supplementary Table S1.

### Self-reported sensory changes were only partially reflected in psychophysical test results during isolation (T1–T3)

**Fig. 4:**
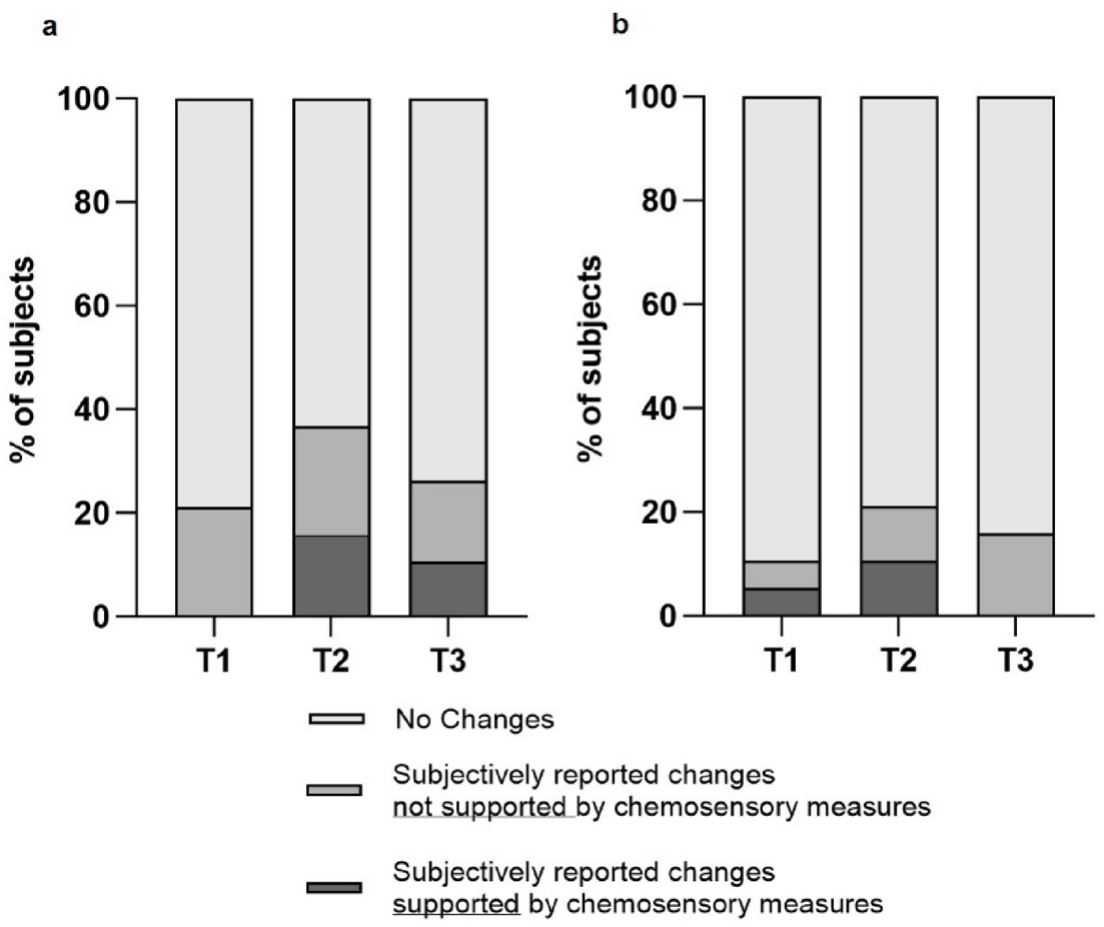
Psychophysical confirmed changes in subjective reports on olfactory and gustatory sensitivity. Frequency of subjects that reported changes in olfactory (**a**) and gustatory (**b**) sensitivity during the Antarctic stay (*T1* = February, *T2* = June, *T3* = October, N = 19) and the proportion of psychophysical confirmed changes based on smell (**a**) and taste (**b**) tests. Results are presented as percentages (%).

During isolation, changes in smell sensitivity were reported by 4/19 (21%), 7/19 (37%), and 5/19 (26%) of participants at T1, T2, and T3, respectively (Fig. 4A), with no significant differences between time points (Cochran’s Q = 1.4, *p* = 0.497). Initially, participants described reduced sensitivity and nasal congestion. By T3, however, three of the five participants mentioned an improvement. Assessing changes in olfactory sensitivity was described as challenging, likely due to the limited presence of odor stimuli during isolation. Self-reports only partially aligned with psychophysical test outcomes: while at T1, none of those reporting reduced smell met the criteria for hyposmia, alignment was observed in 3/7 at T2 and 2/5 at T3. This overall lack of alignment between self-reports and chemosensory tests was also reflected at the level of change scores, as perceived changes in smell sensitivity did not significantly correlate with measured changes in olfactory performance at any time point (Supplementary Table S1).

Self-reported reductions in overall taste sensitivity were noted by 2/19 (11%) of participants at T1, 4/19 (21%) at T2, and 3/19 (16%) at T3 (Fig. 4B), with no significant differences across time points (Cochran’s Q = 1.2, *p* = 0.549). Perceived impairments in taste sensitivity were partially reflected in the psychophysical test results. At T1 and T2, 50% of participants who reported a decline in taste sensitivity were classified as hypogeusic, whereas none of the participants reporting a decline at T3 met the criteria for hypogeusia. However, perceived decline was significantly associated with the magnitude of change in measured salt taste perception, both from T0 to T1 (ρ = − 0.543, *p* = 0.016) and from T2 to T3 (ρ = − 0.499, *p* = 0.030).

Reported smell preferences commonly included nature-related odors (e.g., forest, flowers, rain) and food-related scents (e.g., vanilla, cinnamon) (Supplementary Figure S3a). Nearly half of participants (9/19, 47.4%) reported stable smell preferences across all time points, while the others showed variability or reported no preference at one or more time points. For taste, participants reported preferences for sweet (e.g., chocolate), sour (e.g., citrus fruits), and bitter flavors (e.g., vegetables or soft drinks) at T1, with sour tastes becoming more prominent at T2. Spicy foods were mentioned occasionally. By T3, participants mentioned a general appreciation of fresh fruits. Taste preferences remained stable across time points in most participants (11/19, 57.9%), with only minor variations or occasional missing responses (Supplementary Figure S3b).

## Discussion

The primary objective of this study was to examine the effect of a prolonged stay in an ICE environment in Antarctica on olfactory and gustatory functions.

Hyposmia appeared to increase over time, accompanied by a downward trend in olfactory identification scores, reflecting substantial interindividual variability in sensory responses. While short-term hypoxia has been linked to olfactory changes^24,28,29,37–40^, the gradual impairment observed here suggests additional or delayed effects beyond constant hypobaric hypoxia. Reduced olfactory stimulation, as noted by a participant who reported difficulties in detecting changes due to limited odor exposure during isolation, may also be a contributing factor. This aligns with research indicating that olfactory stimulation is crucial for sustaining the function of the olfactory system ^41,42^. Additionally, consistent reports of nasal congestion and the prolonged exposure to low indoor humidity point to further environmental stressors that may have negatively impacted olfactory function^32^. While hypoxia, sensory deprivation, low humidity, and psychosocial stress are known to influence chemosensory function, the current study design does not allow for causal conclusions. However, the delayed onset of olfactory decline rather suggests that cumulative effects or additional factors may have contributed, though proposed mechanisms remain speculative.

Exploratory analysis six months post-isolation (T4) of five subjects revealed a significant decline in olfactory function compared to baseline, reflected in lower TDI, threshold, and identification scores. In the absence of documented upper respiratory tract infections, these are unlikely to explain the observed decline in olfactory function. Instead, factors such as prolonged sensory deprivation^41,42^ or cumulative in-mission effects^32,33,41,42^ may be more relevant. However, the significant interindividual variability observed at previous time points and the small sample size (n = 5) warrant cautious interpretation.

Partial hypogeusia appeared to increase, primarily reflecting a decline in salty taste sensitivity. The decline became most pronounced toward the end of the mission, suggesting possible long-term adaptation processes rather than acute effects^27^. Specifically, participants with partial hypogeusia showed reduced sensitivity to salty and sour tastes (both mediated by ion channels^43^), whereas their sensitivity to sweet and, to a lesser extent, bitter tastes (mediated by G protein-coupled receptors^43^) appeared to increase. The more pronounced increase in sweet taste perception may reflect an adaptive response to a higher daily caloric demand^16^. This response pattern has not been observed in other short- or long-term ICE studies^44^, and may provide new insight into how prolonged exposure to extreme environments affects taste perception. Concordia’s dry conditions, with low indoor humidity and variable outdoor humidity, may have contributed to these changes. Although there is limited direct evidence linking low humidity to taste changes, prolonged dryness could affect the oral mucosa^45,46^ due to fluid and electrolyte loss, which might influence how salty and sour tastes are perceived.

Additionally, the lack of olfactory stimuli at Concordia may have contributed to changes in taste sensitivity. Hyposmia is known to increase salt consumption^47^, which over time could reduce salt sensitivity^48^. Supporting this, participants with lower salt taste scores at T3 often showed hyposmia, and salty taste scores correlated with TDI scores, suggesting a compensatory increase in salt use^47,48^. After returning home, salt taste sensitivity seemed to normalize, likely due to better diet and environmental conditions. However, the prevalence of partial hypogeusia for sour remained high after isolation. This may be attributed to a combination of factors, including delayed mucosal recovery, limited exposure to acidic stimuli, and taste confusion. Given the many potential confounding factors, the causal relationships cannot be conclusively determined within the scope of this study. Although the sample size was sufficient to detect medium to large effects, further investigation is needed to clarify the underlying mechanisms, including potential sex-specific responses, interindividual variability in response to environmental stressors, and the impact of low test–retest reliability in taste measures^49^.

Olfactory and gustatory impairments did not consistently co-occur, but their correlation at T2 suggests partial interdependence. This aligns with previous findings: Stinton et al.^50^ found no clear effect of smell loss on taste, while Han et al.^51^ observed reduced taste sensitivity in individuals with smell disorders, although inconsistently. Similarly, Landis et al.^52^ reported a moderate decline in taste with long-term smell loss.

Notably, subjective reports of chemosensory impairments were less frequent than the impairments measured in the olfactory and gustatory tests. This limited agreement is well-documented in the literature, both in standard conditions^53^ and in other ICE environments, such as space missions^19,20,30^. Self-reported evaluations of olfaction in healthy, untrained individuals are often considered unreliable, influenced by nasal airflow rather than actual olfactory performance. This may be due to minimal attention typically given to smell in daily life^53^. In addition, subjective assessments of taste function have been shown to lack reliability^35^, with questionnaires^54^ often confirming the absence rather than the presence of impairments^35^. Taste dysfunction diagnosis is challenging due to several factors, including the low prevalence of impairments and misattribution of odor perception deficits to taste issues^54^. However, research during the COVID-19 pandemic showed that self-reports are reliable when impairments are severe or sudden^55,56^. Interestingly, three participants reported heightened smell sensitivity at the end of the Antarctic stay (T3), despite unchanged psychophysical measures, possibly due to odor deprivation enhancing occasional smells during isolation.

Participants frequently named natural odors, which were absent in the low-odor environment^32^, indicating sensory deprivation and a craving for natural stimuli. In several cases, these odors were associated with personal places of origin, suggesting an underlying sense of homesickness^57^. Food-related scents may be associated with comfort, indicating potential emotional coping^58^. Correspondingly, sweet taste preference was elevated early in isolation, possibly reflecting stress^59,60^ or higher energy demands during the active summer phase^61^.

### Strengths and limitations

A key strength of this study is the unique year-long dataset from Concordia Station, collected under standardized ICE conditions. The repeated-measures design with five measurement points, including a baseline and a six-month follow-up, provides rare insights into the long-term progression of olfactory and gustatory functions. Validated tests were transported under controlled conditions and administered by trained crew members, ensuring high-quality results.

While the sample size (N = 19) is small, it exceeds typical ICE study cohorts (median N = 4–10)^62,63^. The predominantly male sample limiting sex-specific analyses, the lack of a control group, the relatively low test–retest reliability of the Taste Strips^49^ and incomplete follow-up data reduce generalizability. However, Bayesian inference and exploratory subgroup analyses helped mitigate some of these limitations. Above-normal baseline prevalence of chemosensory deficits in some participants may be a potential confounder. Causal attribution remains limited, as the study design does not allow for the distinct effects of hypoxia, low humidity, confinement, or sensory deprivation to be disentangled^32^.

## Conclusion

This study suggests that prolonged exposure to an ICE environment, such as overwintering at Concordia Station, may lead to temporary and selective changes in smell and taste, with considerable individual variability. These findings highlight the potential benefit of increasing chemosensory stimulation during extended periods of isolation. While interpretability is limited by the sample size and study design, the findings nevertheless underscore the importance of monitoring chemosensory function in extreme settings—not only in polar and space missions, but also in other isolated or sensory-restricted environments such as submarines, remote work sites, or long-term care. Understanding these changes can help support appetite, mood, and well-being during prolonged confinement.

## Methods

### Study design

The study followed a repeated measures design with data collection at five time points (T0–T4): baseline (T0) six weeks before departure, during the Antarctic stay at the end of summer (February, T1), mid-winter (June, T2), and end of winter (October, T3), and at follow-up six months after return (T4). In line with the European Space Agency (ESA) protocol, this schedule captured long-term effects of the year-long mission at Concordia Station, which included continuous isolation from February to late October/early November. At each time point, standardized taste and smell tests were conducted to assess gustatory and olfactory function. Self-reported evaluations of taste and smell abilities were gathered via questionnaires during the isolation period (T1–T3).

### Study setting

Concordia Station is located on the Antarctic Plateau at Dome C at 3233 m (10,607 ft) above sea level. Due to low barometric pressure, this corresponds to a physiological altitude of approximately 3800 m, resulting in chronic hypobaric hypoxia with inspired oxygen partial pressures at 21% O₂ ranging between 132 and 136 hPa (99.01–102.01 mmHg). The environment is characterized by extreme cold, low humidity, minimal microbial exposure, sensory deprivation, and the absence of natural light during the polar night^32^. Key environmental characteristics are summarized in Table 2. Concordia Station is jointly operated by the French Polar Institute (IPEV) and the Italian Antarctic Program (PNRA) and serves as a multidisciplinary research platform, enabling a wide range of scientific investigations in fields such as astronomy, glaciology, atmospheric sciences, and human physiology. During the Antarctic winter, the station hosts up to 13 crew members and remains completely isolated for nine months. Meals are prepared daily by professional chefs using mostly shelf-stable, frozen, or canned foods, with limited availability of fresh fruits and vegetables. As one of the most remote and environmentally extreme research stations on Earth, Concordia Station serves a validated analog for spaceflight, providing valuable insights into human adaptation in ICE environments and supporting the development of countermeasures to maintain health and performance.

**Table 2:** Overview of ambient environmental conditions at Concordia at overwintering period 2019/2020.

	T1	T2	T3
Outdoor temperature (°C)	− 40.55 ± 5.29	− 69.18 ± 5.41	− 52.85 ± 2.34
Indoor temperature (°C)	23.05 ± 0.88	22.55 ± 1.08	22.64 ± 0.77
Indoor humidity (%)	32.27 ± 0.45	26.77 ± 3.33	25.18 ± 0.65
Outdoor humidity (%)	60.55 ± 5.39	29.91 ± 4.29	42.09 ± 2.25
Barometric pressure (hPa)	649.32 ± 5.38	635.95 ± 6.36	635.48 ± 4.00

Environmental parameters measured by the study physician of overwintering period 2019/2020 during isolation (*T1* = February, *T2* = June, *T3* = October).

### Study population

This study included a convenience sample of 22 Caucasian volunteers (77.3% male; mean age: 38.1 ± 10.7 years, range: 24–56 years), comprising all crew members of the 2019/2020 and 2021/2022 overwintering teams at the Antarctic Concordia research station. No data were collected during the 2020/2021 season due to COVID-19-related operational restrictions. Crew members underwent a rigorous recruitment process coordinated by PNRA and IPEV in collaboration with ESA. The process included medical, physiological, and psychological evaluations, as well as interviews, to confirm participants’ physical fitness and psychological suitability for enduring prolonged isolation and extreme environments. Due to missing baseline data (n = 2) and one dropout after baseline assessment, the final sample consisted of 19 participants (84.2% male; mean age: 39.2 ± 10.9 years). The study was conducted in accordance with the ethical principles of the Declaration of Helsinki and was approved by the Ethics Committee of the University Hospital Tübingen, Germany (NCT03746145). Written informed consent was obtained from all participants prior to enrollment.

### Data collection

The commercial taste and smell test kits were transported to Concordia station under controlled temperature conditions to prevent freezing, ensuring their integrity and reliability. All kits were used within their recommended shelf-life and stored appropriately. According to manufacturer guidelines, repeated use during the study period does not compromise test validity. At each time point, assessments were performed by one of two trained study physicians, each responsible for all repeated measures within a campaign.

Olfactory function was assessed using the validated ODOFIN Sniffin’ Sticks® test (blue kit; Burghart Messtechnik GmbH, Holm, Germany), which measures odor threshold (T, single-staircase procedure), discrimination (D, 16 odorant triplets, three-alternative forced choice), and identification (I, 16 common odorants, four-alternative forced choice). Each subtest has a maximum score of 16, resulting in a combined TDI score ranging from 1 to 48, with higher scores indicating better olfactory performance^64^. Tests were conducted under standardized, postprandial morning conditions. Five distinct randomization protocols were used for threshold and discrimination subtests (Supplementary Table S2) to determine the sequence and position of target pens and to reduce carry-over effects. No randomization was applied for the identification subtest, as participants received no feedback after testing, thereby minimizing potential learning effects across sessions. According to normative data, olfactory function was classified as anosmia (TDI ≤ 16), hyposmia (TDI 16.25–30.5), or normosmia (TDI 30.75–41.25)^34,65^. The assessment protocol and its clinical relevance are described in detail elsewhere^34,66^, highlighting its established use in both clinical and research settings.

Gustatory function was assessed using standardized ODOFIN Taste Strips® (Burghart Messtechnik GmbH, Holm, Germany), representing the four basic taste qualities that were impregnated with four graded concentrations of the respective stimuli (sweet: 0.4, 0.2, 0.1, 0.05 g/mL sucrose; sour: 0.3, 0.165, 0.09, 0.05 g/mL citric acid; salty: 0.25, 0.1, 0.04, 0.016 g/mL sodium chloride; bitter: 0.006, 0.0024, 0.0009, 0.0004 g/mL quinine hydrochloride)^49,67,68^. The test includes 16 impregnated filter paper strips and 2 blanks, presented in randomized order (Supplementary Table S2). Correct identifications were summed to a total taste score (range: 0–16), with subscores (0–4) for each quality. Blank strips were excluded from scoring. The full methodological protocol follows established guidelines^67,68^. According to normative criteria, hypogeusia was defined as a total score below the 10th percentile (< 9 points), and partial hypogeusia as reduced sensitivity to specific taste qualities (sweet, sour, salty < 2; bitter < 1)^68^, allowing more detailed interpretation compared to the olfactory tests. Assessments were performed under preprandial, controlled conditions in the morning, with instructions to abstain from food, beverages (except water), smoking, or oral hygiene one hour prior. Tests were conducted with open nares. This method is validated for adult use and widely applied in both clinical and research settings, as described in detail elsewhere^68–70^.

To complement the psychophysical assessments, participants completed a non-standardized self-report on chemosensory perception several days after psychophysical testing. The use of such self-report measures is a well-established procedure in chemosensory research, particularly for capturing individual sensory experiences that may not be fully reflected in standardized tests^55,71^. The self-reports were obtained several days after the psychophysical testing to minimize immediate test-related bias. In addition to reporting perceived changes in odor and taste preferences, participants were also asked whether they had noticed any alterations in their ability to perceive smells or tastes. Responses were provided in free-text format.

### Statistical analysis

Data analysis was performed using SPSS 28.0.0.0 (SPSS Inc, Chicago, Illinois), JASP 0.18.1 (Intel) (JASP Team, 2023; https://jasp-stats.org/), and GraphPad Prism 10.1.1 for visualization. The main analyses (T0–T3) included 19 participants, with sufficient power (0.81) to detect medium-to-large effects (f = 0.28) via repeated measures F-test (α = 0.05; G*Power 3.1.9.7)^72^. Follow-up data (T4) were available for 5 participants (olfaction) and 15 participants (taste). No relevant baseline differences were found between these subgroups and the full sample.

Data are reported as mean ± SD, and frequencies are given in absolute values and percentages (%). Reference values for dysfunction prevalence were derived using weighted means.

Normally distributed data were analyzed using one-way repeated measures ANOVA, with Greenhouse–Geisser correction applied where appropriate. Non-normally distributed data were analyzed using Friedman tests. For binary variables, Cochran’s Q test was used to evaluate differences over time. Spearman’s ρ was used to calculate correlations between variables, given the ordinal nature of self-ratings and the non-normal distribution of test scores.

Significance was set at *p* < 0.05. Bayesian repeated measures ANOVA (prior width = 0.5) complemented frequentist results. Bayes factors (BF₁₀) quantified evidence strength: BF₁₀ > 1 supports H₁, < 1 supports H₀; values between 1–3 or 0.33–1 indicate weak, 3–10 or 0.1–0.33 moderate, and > 10 or < 0.1 strong evidence, respectively.

## Supplementary Information

Below is the link to the electronic supplementary material.

Supplementary Material 1

## Data Availability

The data that support the findings of this study are available from the corresponding author, IM, upon reasonable request.
