# Supplementary material for: Effects of one year of extreme isolation in Antarctica on olfactory and gustatory functions
Source: Sci Rep. 2025 Sep 5;15:32369. doi: 10.1038/s41598-025-16900-x (PMC12413445; doi:10.1038/s41598-025-16900-x)
Supplement: Supplementary file 1 — Supplementary Material 1 [file 41598_2025_16900_MOESM1_ESM.pdf]

Effects of One Year of Extreme Isolation in Antarctica on Olfactory and Gustatory Functions - Supplementary Material

Bea Klos[1], Sophia Wolf[1], Kathrin Ohla[2], Stijn Thoolen[3,4], Hannes Hagson[3], Paul Enck[1], Isabelle Mack[1*]

[1]Internal Medicine VI, Psychosomatic Medicine and Psychotherapy, University Hospital Tübingen, Tübingen, Germany.
[2]Science & Research, dsm-firmenich, Satigny, Switzerland.
[3]French Polar Institute Paul-Emile Victor, Brest, France - sponsored by the European Space Agency.
[4]Department of Psychiatry, Massachusetts General Hospital, Harvard Medical School, Boston, MA, USA.

*Corresponding author: Isabelle Mack – isabelle.mack@uni-tuebingen.de

Supplementary Figure S1

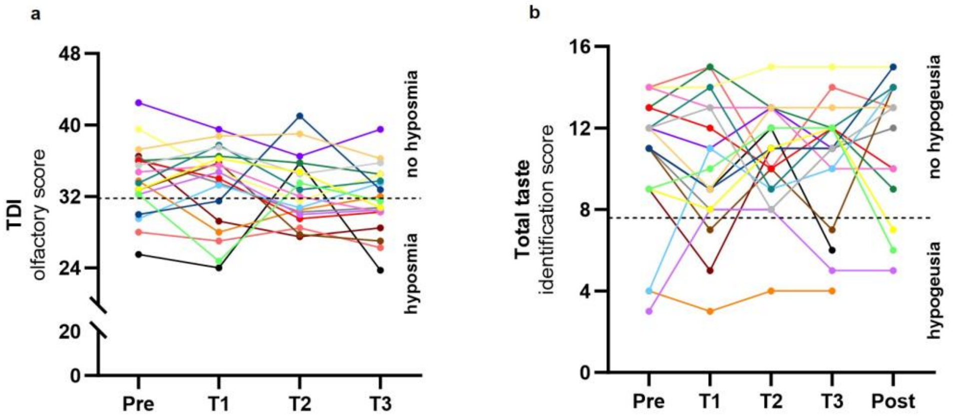

Supplementary Figure S1: Interindividual variability in olfactory and gustatory status across time points.

Interindividual variability in olfactory **[a]** and gustatory **[b]** status before isolation (Pre, n = 19), during isolation (T1 = February, T2 = June, T3 = October, n = 19), and six months post-isolation (Post, n = 15). **[a]** Threshold, Discrimination and Identification (TDI) score range: 0–48; hyposmia: 16.25–30.5; normosmia: ≥ 30.75. T0–T3: Cochran's Q, $p = 0.534$ **[b]** Taste score range: 0–16; hypogeusia: < 9; normogeusia: ≥ 9. T0–T3: Cochran's Q, $p = 0.479$; T0–T4: $p = 0.760$.

Supplementary Figure S2

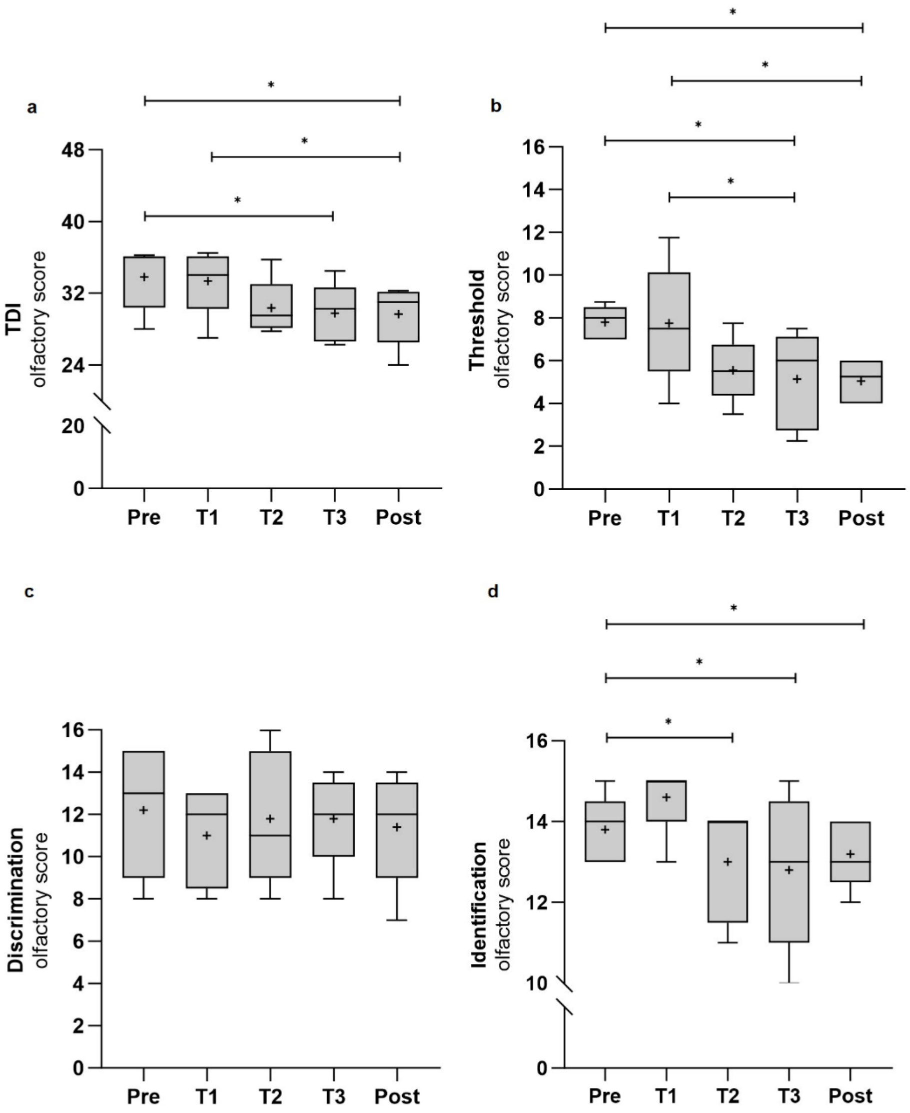

Supplementary Figure S2: Explorative subgroup analysis of olfactory performance of n = 5 across T0-T4.

Test scores for overall smell score (combined threshold, discrimination, and identification (TDI) score **[a]**) and subscores for odor threshold **[b]**, odor discrimination **[c]**, and odor identification **[d]** were assessed before isolation (*Pre*), during isolation (*T1* = February, *T2* = June, *T3* = October), and six months post-isolation (*Post*). Data (n = 19) shown as boxplots: median (—), mean (+), interquartile range (IQR, box), minimum (⊥), and maximum (⊤). Max scores: 16 per subtest, 48 total. Significant differences ($p < 0.05$) marked (*). **[a] TDI score:** ANOVA $p = 0.004$, $BF_{10} = 10.871$ **[b] Threshold**: ANOVA $p = 0.005$, $BF_{10} = 11.904$ **[c] Discrimination:** ANOVA $p = 0.796$ **[d] Identification:** Friedman $p = 0.035$, $BF_{10} = 2.736$.

Supplementary Table S1: Overview of chemosensory performance across timepoints.

	T0 (Pre)	T1 (Feb)	T2 (May)	T3 (Oct)	T4 (Post)	Between-timepoint effects
**Smell test** (M ± SD)	N = 19	N = 19	N = 19	N = 19		
**Threshold**	7.61 ± 2.63	7.07 ± 2.07	6.30 ± 2.70	6.00 ± 2.78	n.a.	n.s
**Discrimination**	11.95 ± 2.55	12.11 ± 2.79	12.79 ± 2.10	11.58 ± 1.84	n.a.	n.s
**Identification**	14.37 ± 1.07	14.11 ± 1.59	13.63 ± 1.16	14.11 ± 1.52	n.a.	***p* = 0.054^{T} (T0 > T3)**
**TDI**	33.92 ± 4.01	33.28 ± 4.64	32.72 ± 3.75	31.68 ± 3.76	n.a.	n.s
**Hyposmia** (%)	21.05%	26.32%	36.84%	36.84%	n.a.	n.s.
**Taste test** (M ± SD)	N = 19	N = 19	N = 19	N = 19	N = 15	
**Sweet**	3.05 ± 1.13	3.16 ± 1.12	3.37 ± 0.68	3.47 ± 0.70	3.47 ± 1.06	n.s
**Sour**	2.11 ± 0.88	2.32 ± 0.82	2.26 ± 0.99	2.21 ± 1.08	2.27 ± 1.10	n.s
**Salty**	2.68 ± 1.29	2.53 ± 1.22	2.26 ± 1.24	2.05 ± 1.08	3.00 ± 1.07	***p* = 0.036* (T2 < T4, T3 < T4)**
**Bitter**	2.58 ± 1.39	2.21 ± 1.44	2.63 ± 1.21	2.74 ± 1.19	2.60 ± 1.45	n.s
**Taste Sum**	10.42 ± 3.39	10.21 ± 3.36	10.53 ± 2.55	10.47 ± 2.95	11.33 ± 3.31	n.s
**Hypogeusia** (%)	15.79%	31.58%	21.05%	21.05%	20.00%	n.s
**Sweet**	15.79%	10.53%	0%	0%	13.34%	n.s
**Sour**	21.05%	15.79%	21.05%	26.32%	33.34%	n.s
**Salty**	15.79%	10.53%	31.58%	31.58%	6.67%	***p* = 0.085^{T} (T0 > T4)**
**Bitter**	15.79%	15.79%	10.53%	5.26%	13.34%	n.s
**Hyposmia** (n) **/ Subnormal Taste Score** (n)						
**Sweet**	1/3	1/2	0/0	0/0	n.a.	n.s.
**Sour**	1/4	2/3	1/4	2/5	n.a.	n.s.
**Salty**	1/3	2/2	4/6	4/6	n.a.	n.s.
**Bitter**	1/3	1/3	2/2	0/1	n.a.	n.s.
**Taste Sum**	1/3	2/4	3/6	3/6	n.a.	n.s.
**TDI score** (ρ)						
**Sweet**	0.169	0.532	0.279	0.112	n.a.	**T1: *p* = 0.019***
**Sour**	0.301	0.101	-0.166	0.079	n.a.	n.s.
**Salty**	0.162	0.328	0.356	0.542	n.a.	**T3: *p* = 0.016***
**Bitter**	0.235	0.001	0.497	0.229	n.a.	**T2: *p* = 0.030***
**Taste Sum**	0.366	0.255	0.479	0.307	n.a.	**T2: *p* = 0.038***
**Threshold score** (ρ)						
**Sweet**	-0.163	0.203	0.036	-0.119	n.a.	n.s.
**Sour**	0.274	0.016	-0.024	-0.029	n.a.	n.s.
**Salty**	-0.036	0.179	0.075	0.258	n.a.	n.s.
**Bitter**	0.119	0.014	0.091	0.035	n.a.	n.s.
**Taste Sum**	0.006	0.098	0.195	0.075	n.a.	n.s.
**Discrimination score** (ρ)						
**Sweet**	0.356	0.685	0.430	0.408	n.a.	**T1: *p* = 0.001***
**Sour**	-0.004	-0.097	-0.212	0.176	n.a.	n.s.
**Salty**	0.294	0.219	0.262	0.306	n.a.	n.s.
**Bitter**	0.242	-0.108	0.596	0.351	n.a.	**T1: *p* = 0.007***
**Taste Sum**	0.476	0.194	0.436	0.315	n.a.	**T0: *p* = 0.039***
**Identification score** (ρ)						
**Sweet**	-0.091	0.241	0.140	0.234	n.a.	n.s.
**Sour**	0.178	0.333	0.057	0.005	n.a.	n.s.
**Salty**	-0.113	0.254	0.193	0.461	n.a.	**T3: *p* = 0.047***.
**Bitter**	-0.191	0.110	0.426	0.063	n.a.	n.s.
**Taste Sum**	-0.099	0.243	0.280	0.152	n.a.	n.s.

**Δ Self-report Taste** (ρ)						
**Δ Sweet**	n.a.	-0.193	-0.248	0.097	n.a.	n.s
**Δ Sour**	n.a.	0.070	0.184	-0.028	n.a.	n.s
**Δ Salty**	n.a.	-0.543	0.254	-0.499	n.a.	**T1: *p* = 0.016*; T3: *p* = 0.030***
**Δ Bitter**	n.a.	< 0.001	0.012	-0.287	n.a.	n.s
**Δ Taste Sum**	n.a.	-0.254	0.214	-0.417	n.a.	n.s
**Δ Self-report Smell** (ρ)						
**Δ Threshold**	n.a.	-0.260	0.240	0.040	n.a.	n.s
**Δ Discrimination**	n.a.	-0.192	0.172	-0.184	n.a.	n.s
**Δ Identification**	n.a.	-0.217	-0.342	-0.071	n.a.	n.s
**Δ TDI**	n.a.	-0.448	0.160	0.043	n.a.	n.s

Notes: Overview of chemosensory test results across five time points (T0: Pre-departure, T1: February, T2: May, T3: October, T4: Follow-up). The table presents mean ± standard deviation (M ± SD) for quantitative olfactory measures (threshold, discrimination, identification, TDI score) and gustatory qualities (sweet, sour, salty, bitter, total taste score) across five time points (T0: pre-departure; T1: February; T2: May; T3: October; T4: follow-up). Frequencies (%) of hyposmia, hypogeusia, and subnormal scores for each taste modality are reported, along with the number of individuals (n) with subnormal taste scores who simultaneously exhibited hyposmia (e.g., 1/3 for sweet). Spearman's rank correlation coefficients (ρ) reflect associations between smell scores and individual taste subscores, as well as between psychophysical test results and changes in self-reported smell and taste perception (Δ self-report). p-values for olfactory and gustatory performance are based on Friedman tests with pairwise post-hoc comparisons. Differences in the prevalence of hyposmia and hypogeusia were analyzed using Cochran's Q test. Statistically significant effects ($p < .05$) are marked with an asterisk (*), and trends ($0.05 \leq p < .10$) with a "T". Abbreviations: n.a. = not applicable; n.s. = not significant.

Supplementary Figure S3

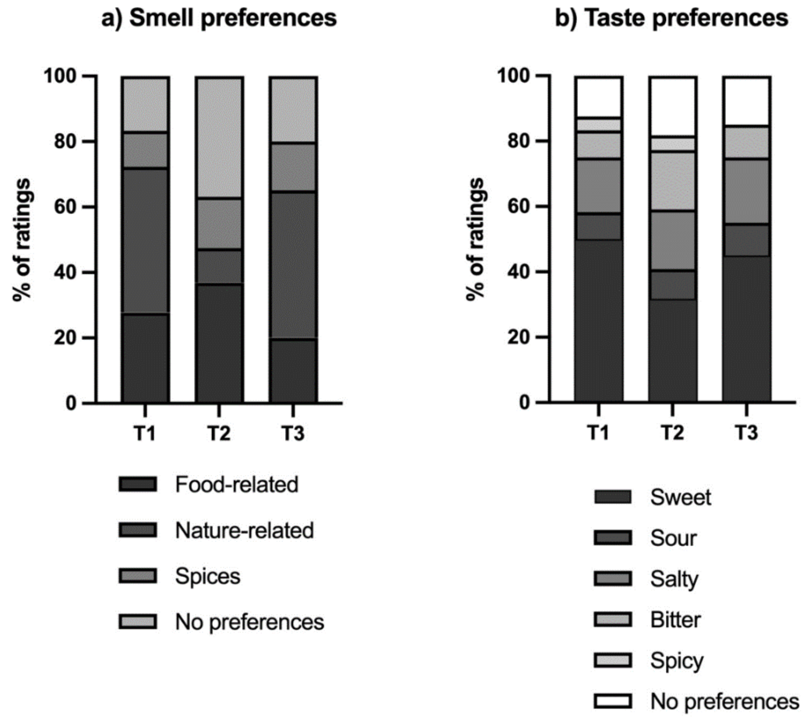

Supplementary Figure S3: Smell and taste preferences during isolation (T1-T3).

[a] Self-reported smell preferences at three time points during the mission (T1–T3). Preferences were grouped into food-related (including sweets, fruits, bread and cakes, meat/hamburger, garlic), nature-related (including forest, sea, mountains, salty air, flowers, (burned) wood/smoke, and homeland fields) and spice-related categories (including vanilla and cinnamon); "no preferences" indicates participants who did not report any specific preference. Values represent the proportion of total responses per category and time point. **[b]** Self-reported taste preferences during the mission (T1–T3). Values represent the proportion of total responses per category and time point. "No preferences" indicates participants who did not report any specific preference.

Supplementary Table S2: Randomization protocol.

A. Smell – Threshold test

ID	T0	T1	T2	T3	T4
A	Sequence 4	Sequence 1	Sequence 2	Sequence 3	Sequence 5
B	Sequence 3	Sequence 5	Sequence 1	Sequence 2	Sequence 4
C	Sequence 2	Sequence 3	Sequence 1	Sequence 4	Sequence 5
D	Sequence 2	Sequence 1	Sequence 3	Sequence 4	Sequence 5
E	Sequence 5	Sequence 4	Sequence 3	Sequence 2	Sequence 1
F	Sequence 2	Sequence 4	Sequence 3	Sequence 2	Sequence 1
G	Sequence 1	Sequence 5	Sequence 2	Sequence 3	Sequence 4
H	Sequence 1	Sequence 4	Sequence 3	Sequence 2	Sequence 5
I	Sequence 1	Sequence 2	Sequence 3	Sequence 4	Sequence 5
K	Sequence 4	Sequence 3	Sequence 2	Sequence 1	Sequence 5
L	Sequence 2	Sequence 5	Sequence 1	Sequence 3	Sequence 4
M	Sequence 3	Sequence 4	Sequence 5	Sequence 2	Sequence 1
N	Sequence 5	Sequence 3	Sequence 4	Sequence 1	Sequence 2

Sequence 1:

step	turning point 1	turning point 2	turning point 3	turning point 4	turning point 5	turning point 6	turning point 7
1	1,1	2,2	2,2	2,3	3,3	3,3	2,3
2	2,1	3,1	2,2	1,1	2,1	1,2	3,3
3	3,1	1,1	1,2	2,1	1,2	2,1	1,2
4	3,2	2,1	3,1	3,2	2,3	3,1	1,1
5	1,2	3,2	1,1	1,2	1,1	1,3	2,1
6	2,3	2,3	3,2	3,3	2,2	3,3	2,3
7	3,1	1,2	3,3	1,3	3,1	1,3	1,3
8	1,3	2,1	1,3	3,1	1,3	2,1	2,3
9	2,1	3,1	2,1	1,1	2,3	2,2	2,2
10	1,2	2,2	2,2	2,2	3,2	1,2	1,1
11	3,2	1,1	3,1	2,3	1,3	2,3	2,1
12	2,2	3,3	1,2	3,2	3,2	3,2	3,1
13	3,3	2,1	3,2	1,2	2,3	1,1	1,2
14	1,2	1,3	3,3	3,3	3,3	2,2	3,2
15	1,1	3,2	1,3	1,1	1,3	2,3	1,3
16	2,1	3,3	2,2	3,3	3,1	3,1	2,2

Sequence 2:

step	turning point 1	turning point 2	turning point 3	turning point 4	turning point 5	turning point 6	turning point 7
1	2,2	3,3	1,2	3,1	3,1	3,1	3,1
2	1,1	2,2	2,3	1,2	2,2	1,3	1,1
3	3,3	1,1	3,3	3,1	1,3	2,1	2,1
4	2,3	3,3	1,2	1,2	2,3	3,2	3,2
5	1,1	3,2	2,3	2,3	3,3	1,1	1,3
6	2,2	1,1	3,2	3,2	3,1	1,3	2,1
7	3,3	2,2	2,3	3,1	1,3	2,1	1,3
8	2,2	1,1	1,2	1,2	2,3	1,3	3,2
9	1,1	3,3	3,2	2,3	3,1	3,2	2,1
10	3,3	2,2	3,2	3,2	1,3	2,1	1,3
11	2,2	1,1	1,2	1,2	3,1	3,1	3,2
12	3,3	3,2	2,3	3,2	2,3	1,3	2,1
13	1,1	2,1	3,2	2,2	1,3	3,2	1,2
14	2,2	1,1	1,2	3,1	3,1	2,1	3,2
15	3,3	2,2	2,3	1,3	2,2	3,1	2,1
16	3,3	3,2	3,2	3,1	3,2	1,2	1,1

Sequence 3:

step	turning point 1	turning point 2	turning point 3	turning point 4	turning point 5	turning point 6	turning point 7
1	1,1	3,3	2,2	1,3	3,1	2,1	2,3
2	2,1	1,1	3,3	2,2	1,3	3,1	3,1
3	3,2	2,1	1,1	3,3	2,2	1,3	1,3
4	2,3	3,2	2,1	1,1	3,3	2,2	2,2
5	1,2	2,3	3,2	2,1	1,1	3,3	3,3
6	2,1	1,2	2,3	3,2	2,1	1,1	2,2
7	3,3	2,1	1,2	2,3	3,2	2,1	1,1
8	2,2	3,3	2,1	1,2	2,3	1,1	2,1
9	1,3	2,2	3,3	2,1	1,1	2,1	3,2
10	3,1	1,3	2,2	1,1	2,1	3,2	2,3
11	2,1	3,1	1,1	2,1	3,2	2,3	1,2
12	3,1	1,1	2,1	3,2	2,3	1,2	2,1
13	1,1	2,1	3,2	2,3	1,2	2,1	3,3
14	2,1	3,2	2,3	1,2	2,1	3,3	2,2
15	3,2	2,3	1,2	2,1	3,3	2,2	1,3
16	2,3	1,2	2,1	3,3	2,2	1,3	3,1

Sequence 4:

step	turning point 1	turning point 2	turning point 3	turning point 4	turning point 5	turning point 6	turning point 7
1	1,1	2,3	3,1	2,2	1,3	3,2	1,3
2	2,1	1,1	2,3	3,1	2,2	1,3	3,2
3	3,2	2,1	1,1	2,3	3,1	2,2	1,3
4	1,2	3,2	2,1	1,1	2,3	3,1	2,2
5	2,3	1,2	3,2	2,1	1,1	2,3	3,1
6	3,1	2,3	1,2	3,2	2,1	1,1	2,3
7	2,2	3,1	2,3	1,2	3,2	2,1	1,1
8	1,3	2,2	3,1	2,3	1,2	3,2	2,1
9	3,1	1,3	2,2	3,1	2,3	1,2	3,2
10	3,2	3,1	1,3	2,2	3,1	2,3	1,2
11	2,1	3,2	3,1	1,3	2,2	3,1	2,3
12	1,1	2,1	3,2	3,1	1,3	2,2	3,1
13	2,2	1,1	2,1	3,2	3,1	1,3	2,2
14	3,1	3,1	1,1	2,1	3,2	3,1	1,3
15	2,1	2,1	3,2	1,1	2,1	3,2	3,1
16	3,1	1,1	2,2	3,1	1,1	1,1	3,2

Sequence 5:

step	turning point 1	turning point 2	turning point 3	turning point 4	turning point 5	turning point 6	turning point 7
1	3,1	2,1	1,2	3,2	3,2	3,3	2,2
2	1,1	1,3	2,1	1,1	1,3	1,1	1,3
3	2,1	2,2	1,2	2,1	2,2	2,1	3,2
4	3,1	3,1	3,1	3,1	3,1	3,1	3,1
5	1,2	1,1	2,2	1,3	2,1	1,3	1,1
6	2,2	2,1	1,3	2,2	1,2	2,2	2,1
7	3,2	3,1	3,2	3,1	3,1	3,1	3,1
8	2,2	1,2	1,1	2,1	2,2	2,1	1,2
9	1,3	2,2	2,1	1,2	1,3	1,2	2,2
10	3,2	3,2	3,1	3,1	3,2	3,1	3,2
11	2,1	2,2	1,2	1,1	1,2	2,2	2,2
12	1,2	1,3	2,2	2,1	2,2	1,3	1,3
13	3,1	3,2	3,2	3,1	3,2	3,2	3,2
14	1,3	2,1	2,2	2,2	1,1	1,2	2,1
15	2,2	1,2	1,3	1,3	2,1	2,2	1,2
16	3,1	3,1	3,2	3,2	3,1	3,2	3,1

Note: Randomization protocol of the olfactory threshold test: first digit indicates the position of the target pen in the first trial of each step, second digit indicates the position in the second trial.

B. Smell – Discrimination test

ID	T0	T1	T2	T3	T4
A	Sequence 1	Sequence 2	Sequence 3	Sequence 4	Sequence 5
B	Sequence 5	Sequence 4	Sequence 3	Sequence 2	Sequence 1
C	Sequence 2	Sequence 4	Sequence 3	Sequence 2	Sequence 1
D	Sequence 5	Sequence 3	Sequence 4	Sequence 1	Sequence 2
E	Sequence 1	Sequence 5	Sequence 2	Sequence 3	Sequence 4
F	Sequence 4	Sequence 3	Sequence 2	Sequence 1	Sequence 5
G	Sequence 2	Sequence 1	Sequence 3	Sequence 4	Sequence 5
H	Sequence 1	Sequence 4	Sequence 3	Sequence 2	Sequence 5
I	Sequence 3	Sequence 4	Sequence 5	Sequence 2	Sequence 1
K	Sequence 2	Sequence 3	Sequence 1	Sequence 4	Sequence 5
L	Sequence 2	Sequence 5	Sequence 1	Sequence 3	Sequence 4
M	Sequence 4	Sequence 1	Sequence 2	Sequence 3	Sequence 5
N	Sequence 3	Sequence 5	Sequence 1	Sequence 2	Sequence 4

Sequence 1: 1 – 2 – 3 – 3 – 2 – 1 – 1 – 2 – 2 – 3 – 3 – 2 – 3 – 1 – 2 – 3

Sequence 2: 3 – 2 – 1 – 1 – 2 – 3 – 2 – 1 – 1 – 3 – 3 – 3 – 2 – 1 – 3 – 1

Sequence 3: 2 – 2 – 1 – 1 – 3 – 1 – 2 – 3 – 1 – 2 – 3 – 3 – 1 – 2 – 2 – 3

Sequence 4: 1 – 1 – 2 – 2 – 1 – 3 – 1 – 2 – 3 – 1 – 2 – 2 – 3 – 1 – 2 – 1

Sequence 5: 3 – 1 – 3 – 2 – 2 – 3 – 2 – 2 – 1 – 1 – 1 – 3 – 2 – 3 – 1 – 3

Note: Randomization protocol of the olfactory discrimination test: digit indicates the position of the target pen in each trial.

C. Taste Strips

ID	T0	T1	T2	T3	T4
A	Sequence 5	Sequence 3	Sequence 4	Sequence 2	Sequence 1
B	Sequence 2	Sequence 1	Sequence 5	Sequence 4	Sequence 3
C	Sequence 5	Sequence 2	Sequence 1	Sequence 3	Sequence 4
D	Sequence 1	Sequence 5	Sequence 2	Sequence 3	Sequence 4
E	Sequence 3	Sequence 4	Sequence 5	Sequence 2	Sequence 1
F	Sequence 2	Sequence 1	Sequence 3	Sequence 4	Sequence 5
G	Sequence 5	Sequence 4	Sequence 3	Sequence 2	Sequence 1
H	Sequence 3	Sequence 5	Sequence 1	Sequence 2	Sequence 4
I	Sequence 4	Sequence 3	Sequence 2	Sequence 1	Sequence 5
K	Sequence 1	Sequence 3	Sequence 5	Sequence 4	Sequence 2
L	Sequence 5	Sequence 3	Sequence 4	Sequence 1	Sequence 2
M	Sequence 1	Sequence 4	Sequence 3	Sequence 2	Sequence 5
N	Sequence 4	Sequence 2	Sequence 3	Sequence 1	Sequence 5

Sequence 1: Sucrose 0.05 g/mL – Quinine-hydrochloride 0.0004 g/mL – Sodium chloride 0.016 g/mL – Citric acid 0.05 g/mL – Citric acid 0.09 g/mL – Sucrose 0.1 g/mL – *Blank* – Quinine-hydrochloride 0.0009 g/mL – Sodium chloride 0.04 g/mL – Citric acid 0.165 g/mL – Sucrose 0.2 g/mL – Sodium chloride 0.1 g/mL – Quinine-hydrochloride 0.0024 g/mL – *Blank* – Sucrose 0.4 g/mL – Citric acid 0.3 g/mL – Sodium chloride 0.25 g/mL – Quinine-hydrochloride 0.006 g/mL

Sequence 2: Citric acid 0.05 g/mL – Quinine-hydrochloride 0.0004 g/mL – Sodium chloride 0.016 g/mL – Sucrose 0.05 g/mL – Sodium chloride 0.04 g/mL – *Blank* – Sucrose 0.1 g/mL – Citric acid 0.09 g/mL – Quinine-hydrochloride 0.0009 g/mL – Sucrose 0.2 g/mL – Citric acid 0.165 g/mL – Sodium chloride 0.1 g/mL – *Blank* – Quinine-hydrochloride 0.0024 g/mL – Citric acid 0.3 g/mL – Sucrose 0.4 g/mL – Sodium chloride 0.25 g/mL – Quinine-hydrochloride 0.006 g/mL

Sequence 3: Quinine-hydrochloride 0.0004 g/mL – Sodium chloride 0.016 g/mL – Sucrose 0.05 g/mL – Citric acid 0.05 g/mL – *Blank* – Citric acid 0.09 g/mL – Quinine-hydrochloride 0.0009 g/mL – Sucrose 0.1 g/mL –

Sodium chloride 0.04 g/mL – Sodium chloride 0.1 g/mL – Quinine-hydrochloride 0.0024 g/mL – *Blank* – Sucrose 0.2 g/mL – Citric acid 0.165 g/mL – Sodium chloride 0.25 g/mL – Quinine-hydrochloride 0.006 g/mL – Sucrose 0.4 g/mL – Citric acid 0.3 g/mL

Sequence 4: Sodium chloride 0.016 g/mL – Sucrose 0.05 g/mL – Quinine-hydrochloride 0.0004 g/mL – *Blank* – Citric acid 0.05 g/mL – Quinine-hydrochloride 0.0009 g/mL – Sodium chloride 0.04 g/mL – Citric acid 0.09 g/mL – Sucrose 0.1 g/mL – Quinine-hydrochloride 0.0024 g/mL – *Blank* – Citric acid 0.165 g/mL – Sodium chloride 0.1 g/mL – Sucrose 0.2 g/mL – Sodium chloride 0.25 g/mL – Citric acid 0.3 g/mL – Quinine-hydrochloride 0.006 g/mL – Sucrose 0.4 g/mL

Sequence 5: *Blank* – Sodium chloride 0.016 g/mL – Citric acid 0.05 g/mL – Quinine-hydrochloride 0.0004 g/mL – Sucrose 0.05 g/mL – Sodium chloride 0.04 g/mL – Quinine-hydrochloride 0.0009 g/mL – Sucrose 0.1 g/mL – Citric acid 0.09 g/mL – *Blank* – Sodium chloride 0.1 g/mL – Sucrose 0.2 g/mL – Citric acid 0.165 g/mL – Quinine-hydrochloride 0.0024 g/mL – Quinine-hydrochloride 0.006 g/mL – Sucrose 0.4 g/mL – Citric acid 0.3 g/mL – Sodium chloride 0.25 g/mL
